# Etanercept-Synthesising Mesenchymal Stem Cells Efficiently Ameliorate Collagen-Induced Arthritis

**DOI:** 10.1038/srep39593

**Published:** 2017-01-13

**Authors:** Narae Park, Yeri Alice Rim, Hyerin Jung, Juryun Kim, Hyoju Yi, Youngkyun Kim, Yeonsue Jang, Seung Min Jung, Jennifer Lee, Seung-Ki Kwok, Sung-Hwan Park, Ji Hyeon Ju

**Affiliations:** 1 Division of Rheumatology, Department of Internal Medicine, Seoul St. Mary’s Hospital, College of Medicine, The Catholic University of Korea, Seoul, 137-701, Republic of Korea; 2 Department of Medicine, Institute for Stem Cell Biology and Regenerative Medicine, and Stanford Cardiovascular Institute, Stanford University School of Medicine, Stanford, CA, USA

## Abstract

Mesenchymal stem cells (MSCs) have multiple properties including anti-inflammatory and immunomodulatory effects in various disease models and clinical treatments. These beneficial effects, however, are sometimes inconsistent and unpredictable. For wider and proper application, scientists sought to improve MSC functions by engineering. We aimed to invent a novel method to produce synthetic biological drugs from engineered MSCs. We investigated the anti-arthritic effect of engineered MSCs in a collagen-induced arthritis (CIA) model. Biologics such as etanercept are the most successful drugs used in anti-cytokine therapy. Biologics are made of protein components, and thus can be theoretically produced from cells including MSCs. MSCs were transfected with recombinant minicircles encoding etanercept (trade name, Enbrel), which is a tumour necrosis factor α blocker currently used to treat rheumatoid arthritis. We confirmed minicircle expression in MSCs *in vitro* based on GFP. Etanercept production was verified from the conditioned media. We confirmed that self-reproduced etanercept was biologically active *in vitro*. Arthritis subsided more efficiently in CIA mice injected with mcTNFR2MSCs than in those injected with conventional MSCs or etanercept only. Although this novel strategy is in a very early conceptual stage, it seems to represent a potential alternative method for the delivery of biologics and engineering MSCs.

Tumour necrosis factor (TNF) α plays a critical role in the pathogenesis of rheumatoid arthritis (RA). Therefore, the development of a TNFα inhibitor has been vigorously pursued as an attractive treatment for RA^1^,^2^. Etanercept (Enbrel) is one of the most successful biological drugs against TNFα^3^,^4^. Etanercept is a synthetic protein drug that conjoins the p75 region of TNF receptor 2 (TNFR2) with the Fc portion of immunoglobulin^5^,^6^,^7^. Biological drugs are usually generated *in vitro*; however, they are some of the most expensive drugs in the market, which increases medical expenses for treatment^8^. The high cost is due to the recombination and purification processes required for human clinical applications^9^. Therefore, the search for an alternative treatment is ongoing.

Theoretically, biological drugs can also be produced *in vivo* because they are proteins formulated via translation and transcription of DNA sequences. In our previous studies, we attempted to elucidate a new method to deliver a biological drug into a living organism by delivering the DNA sequences encoding etanercept and abatacept^9^,^10^. Using minicircles encoding biological drugs, we confirmed the anti-inflammatory and immunosuppressive effects *in vitro* and *in vivo*. Minicircles are relatively small episomal vectors because the bacterial backbones are removed after gene amplification. The absence of the bacterial backbone and the non-integrating transfection system make minicircle vectors an attractive *in vivo* delivery tool for future clinical applications^11^. Minicircles are thought to be an ideal vehicle to transfer a gene of interest both *in vitro* and *in vivo* and have already been used in preclinical gene therapy research^12^,^13^. Minicircles were used in those attempts because their small size makes transfection more feasible and efficient without integration and the proteins they encode can be secreted *in vivo* with fewer safety issues^11^,^12^,^14^,^15^.

Mesenchymal stem cells (MSCs) are adult stem cells that have immunosuppressive effects against various autoimmune diseases including RA^16^,^17^,^18^. MSCs also have the ability to reconstruct damaged bone and cartilage^19^,^20^,^21^,^22^. MSCs may become a central material for regenerative medicine due to their immunomodulatory and anti-inflammatory effects and regenerative potency^23^,^24^. Although MSCs have several beneficial effects, some researchers claim that these effects are inconsistent and controversial^25^. Scientists are attempting to engineer MSCs in order to improve their function in various ways. It was reported that MSCs engineered with specific genes show improved curative effects^26^,^27^,^28^. To date, it remains difficult to transfer a foreign gene into MSCs^24^. Viruses are the main means by which genes are transferred into MSCs. However, the use of viruses can induce the integration of foreign genes, which is another hurdle for clinical applications. Therefore, an improved method to deliver a foreign gene into MSCs is required. Efficacy and safety are the main issues that need to be resolved for MSC engineering by gene transfer for clinical applications.

In this study, we attempted to generate engineered MSCs named mcTNFR2MSCs using a novel strategy. We generated a unique minicircle plasmid encoding etanercept (mcTNFR2). Using electroporation, we transfected MSCs with mcTNFR2 (mcTNFR2MSC). The generated mcTNFR2MSCs successfully produced etanercept as intended. mcTNFR2MSCs were superior at suppressing arthritis compared with conventional MSCs. We hope that the generation of mcTNFR2MSCs using minicircle vectors can make MSC-based treatment more applicable in the future.

## Results

### Scheme of the experimental concept and drug expression of the generated mcTNFR2

**Figure 1:**
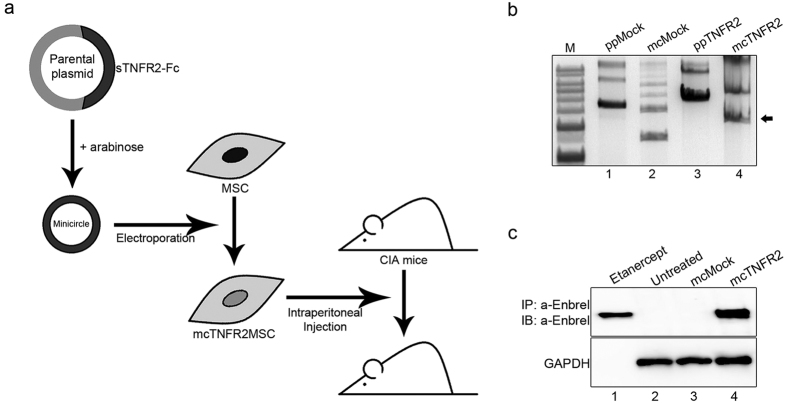
Scheme of the experimental concept and drug expression of the generated mcTNFR2. (**a**) Schematic diagram of experiments using mcTNFR2MSCs in a CIA mouse model. The concept is to produce synthetic biological drugs from mcTNFR2MSCs and to investigate the anti-arthritic effect of mcTNFR2MSCs in a CIA mouse model. (**b**) The representative gel image of parental and minicircle plasmids (mock and sTNFR2). Minicircles were produced by removing the bacterial backbone from the parental plasmid with arabinose. (**c**) Representative Western blot image and analysis to detect sTNFR2-Fc (n = 4). sTNFR2-Fc was detected in the conditioned media of mcTNFR2-transfected HEK293T cells. Etanercept-treated and untreated HEK293T cells were used as controls.

We designed and conducted our experiments following the concept showed in Fig. 1a. We generated mcTNFR2 by arabinose treatment. Then, the generated mcTNFR2 vector was transfected into MSCs to generate mcTNFR2MSCs. mcTNFR2MSCs were delivered to collagen-induced arthritis (CIA) mouse model by intraperitoneal injections to investigate the therapeutic effects on RA. First, minicircle production from parental plasmids was confirmed by gel electrophoresis (Fig. 1b). The generated mcTNFR2 had a size of 3 kb, almost half that of the parental plasmid. To confirm that the produced mcTNFR2 vector can synthesise and secrete soluble TNFR2-Fc protein (sTNFR2-Fc), human embryonic kidney 293 (HEK293T) cells were transfected with mcTNFR2 (mcTNFR2-293T). The isolated and concentrated sTNFR2-Fc was detected by immunoblotting (Fig. 1c). sTNFR2-Fc was strongly detected in the culture supernatant of mcTNFR2-transfected HEK 293 T cells(mcTNFR2-293T), but not in untreated cells or cells transfected with mock minicircle vectors (mcMock-293T). The amount of secreted drug was 1.8-fold higher than the known amount of commercial etanercept (250 ng/mL). We speculated that 231.25 ng/mL sTNFR2-Fc can be secreted per 5 × 10^6^ cells. In summary, we generated mcTNFR2, and cells transfected with this vector synthesised the protein drug sTNFR2-Fc. The full-length gels and blots are included in supplements (Supplementary Fig. S1).

### Generation of mcTNFR2MSCs by transfection with mcTNFR2

**Figure 2:**
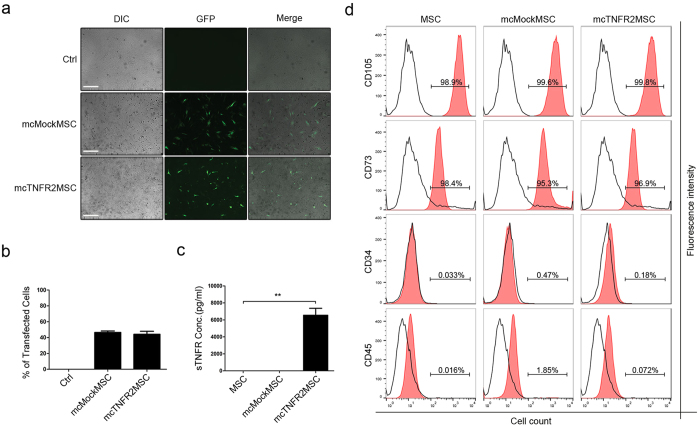
Generation of mcTNFR2MSCs by transfection with mcTNFR2. (**a**) Fluorescence images showing the expression of GFP at 48 h after transfection. Expression of GFP was similar in mcMockMSCs and mcTNFR2MSCs. Experiment was repeated 4–6 times and the representative image is shown. Scale bars: 500 μm. (**b**) The transfection efficacy of mcMock and mcTNFR2 was measured by counting GFP+ cells. (**c**) Concentration of sTNFR2-Fc in the conditioned media of MSCs transfected with minicircles encoding sTNFR2-Fc (mean ± SEM). The conditioned media were removed 48 h post-transfection and analysed by an ELISA. The experiment was repeated three times with each conditioned media. (**d**) Characterisation of mcTNFR2MSCs. The percentage of cells expressing each MSC marker was analysed. The analysed plots represent the fluorescence intensity and cell number per fluorescence channel. Negative control (isotype control) fluorescence is plotted on each panel as a black line. CD105-PE-Cy7 and CD73-FITC were used as positive markers. CD34-APC and CD45-PE were used as negative markers. Each histogram is a representative result of at least three MSC, mcMockMSC and mcTNFR2MSC samples (*P < 0.01; **P < 0.005; ***P < 0.001).

We verified that mcTNFR2 was functional in HEK293T cells; therefore, we attempted to transfect this vector into MSCs. To confirm the transfection efficacy of MSCs, mcMock and mcTNFR2 were transfected (Fig. 2a). GFP expression in MSCs transfected with mcTNFR2 (mcTNFR2MSCs) was similar to that in MSCs transfected with mcMock (mcMockMSCs). The transfection efficacy was higher for mcMockMSCs, but the overall transfection rate of mcTNFR2 was similar to that of mcMock (Fig. 2b). We examined if the transfected mcTNFR2MSCs could synthesise the protein drug *in vitro* (Fig. 2c). sTNFR2-Fc was highly detected in the culture supernatant of mcTNFR2MSCs; however, it was not detected in the culture supernatant of non-transfected MSCs or mcMockMSCs. After we confirmed the transfection of mcTNFR2 and expression of sTNFR2-Fc in mcTNFR2MSCs, we sought to confirm that transfection of a foreign gene or secretion of a protein drug did not disrupt the MSC phenotype. Expression of positive markers of MSCs (CD73 and CD105) in mcMockMSCs and mcTNFR2MSCs was similar to that in non-transfected MSCs (Fig. 2d). The levels of negative markers of MSCs (CD34 and CD45) were also similar to those in non-transfected MSCs. By analysing MSC marker expression, we confirmed that transfection and drug expression did not alter the MSC characteristics of mcTNFR2MSCs. Consequently, we confirmed that mcTNFR2MSCs were successfully generated and were able to maintain the characteristics of wild-type (WT) MSCs throughout the entire process. The generated mcTNFR2MSCs were also able to secrete the desired protein drug, sTNFR2-Fc, *in vitro*.

### Functional analysis of the protein drug sTNFR2-Fc derived from mcTNFR2MSCs *in vitro*

**Figure 3:**
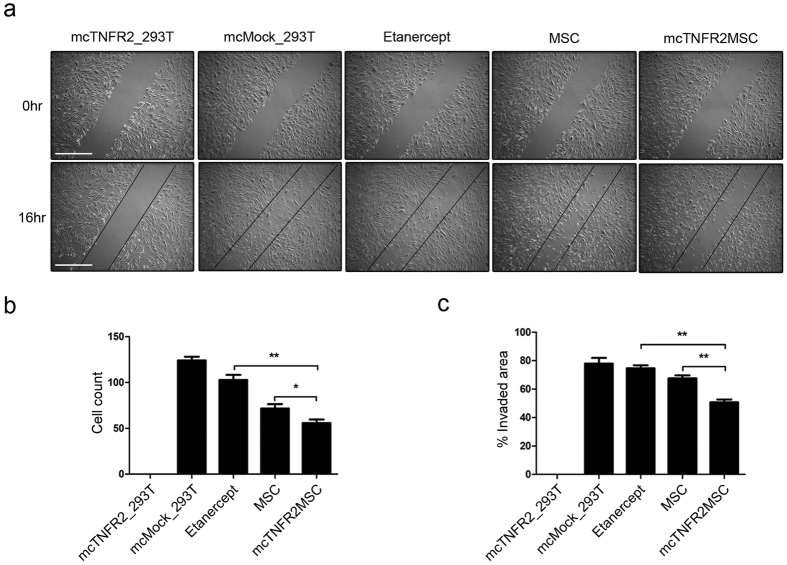
Function of the protein drug sTNFR2-Fc derived from mcTNFR2MSCs *in vitro*. (**a**) Representative microscopy image from scratch assay (n = 3). RA-FLS monolayers were scratched with a sterile pipette tip. Each well was treated with conditioned media of MSCs and HEK293T cells transfected with minicircles. Etanercept was used as a control. Phase-contrast microscopy images were acquired at 0 and 16 h after wounds were created. (**b**) Cell count of wound-invading cells. The number of invading RA-FLS cells was significantly higher in samples treated with the medium control and etanercept. (**c**) Percentage of invaded area. The tendency to migrate was calculated according to the following equation: percentage of invaded area = (1 − (wounded area at 16 h)/(wounded area at 0 h)) × 100. This statistical data represents the results of three individual experiments. (*P < 0.01; **P < 0.005; ***P < 0.001.)

RA joints are characterised by hyperplasia of fibroblast-like synoviocytes (FLSs), which is thought to be promoted by TNFα^29^. We verified the anti-migratory effect of sTNFR2-Fc secreted by mcTNFR2MSCs by performing scratch assay. Migration of RA-FLSs was observed upon TNFα stimulation. The migration of FLSs was significantly decreased by treatment with the culture media of mcTNFR2MSCs (Fig. 3a). The migration of FLSs treated with the culture media of non-engineered MSCs or commercial etanercept was also decreased, but not as much as that of FLSs treated with the culture media of mcTNFR2MSCs. The migration rate was measured (Fig. 3b). The number of migrating FLSs was highly increased in the negative control. The number of migrating FLSs was remarkably decreased upon treatment with the culture media of mcTNFR2MSCs. The invaded area in the scratch was measured. FLSs treated with the culture media of mcTNFR2MSCs failed to fill the scratched area compared with FLSs in the other conditions (Fig. 3c). In conclusion, we confirmed that mcTNFR2MSCs have superior suppressive effects on the migration of RA-FLSs compared with commercial etanercept and non-engineered MSCs.

### *In vivo* injection of mcTNFR2MSCs inhibits the development of arthritis in CIA mice

**Figure 4:**
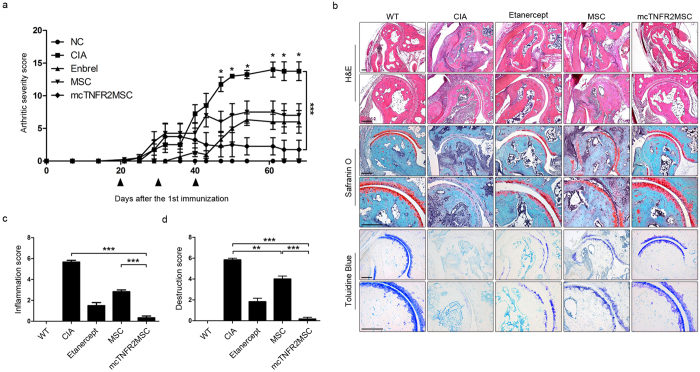
Injection of mcTNFR2MSCs inhibits the development of arthritis in CIA mice. (**a**) Arthritis severity score of mice (n = 5 per group). MSCs and mcTNFR2MSCs were intraperitoneally injected three times. The arrows indicate the day of injection. (**b**) The representative histological images of cartilage and bone erosion. Joints were stained with H&E, safranin O and toluidine blue. Scale bars: 200 μm. (**c**) Inflammation score of histological data. The score was based on the extent of synovial hyperplasia and infiltration of leukocytes. (**d**) Joint destruction scores were based on cartilage loss estimated in samples stained with safranin O and toluidine blue as well as the extent of pannus formation. (*P < 0.01; **P < 0.005; ***P < 0.001).

To investigate the effect of mcTNFR2MSCs *in vivo*, we injected CIA mice with mcTNFR2MSCs. After day 40, the arthritic severity score of CIA mice injected with mcTNFR2MSCs started to decrease and was continuously lower than in the other groups (Fig. 4a). The effect of mcTNFR2MSCs was histologically demonstrated by markedly decreased synovial hypertrophy in joints (Fig. 4b). Haematoxylin and eosin (H&E) staining showed diffuse cellular infiltration and pannus invasion in adjoining bone structures. CIA control mice showed massive damage in the tarsal bones. Joints in the mcTNFR2MSC-injected group showed less infiltration and invasion of the pannus compared with CIA control joints. Cartilage damage was confirmed by safranin O and toluidine blue staining. The level of cartilage damage was lower in the group injected with mcTNFR2MSCs than in the CIA control group. Joints in the MSC-injected group showed less cellular infiltration than those in the CIA control group; however, cartilage damage was similar in these two groups. The inflammation score was significantly lower in the mcTNFR2MSC-injected group than in the other groups (Fig. 4c). The joint destruction score was also significantly lower in the mcTNFR2MSC-injected group, while the joint destruction score in the MSC-injected group was about half of that in the CIA control group (Fig. 4d). These results confirmed that injection of mcTNFR2MSCs could reduce the symptoms of arthritis and that the therapeutic effect of mcTNFR2MSCs was better than that of non-engineered WT MSCs.

### *In vivo* detection of mcTNFR2MSCs

**Figure 5:**
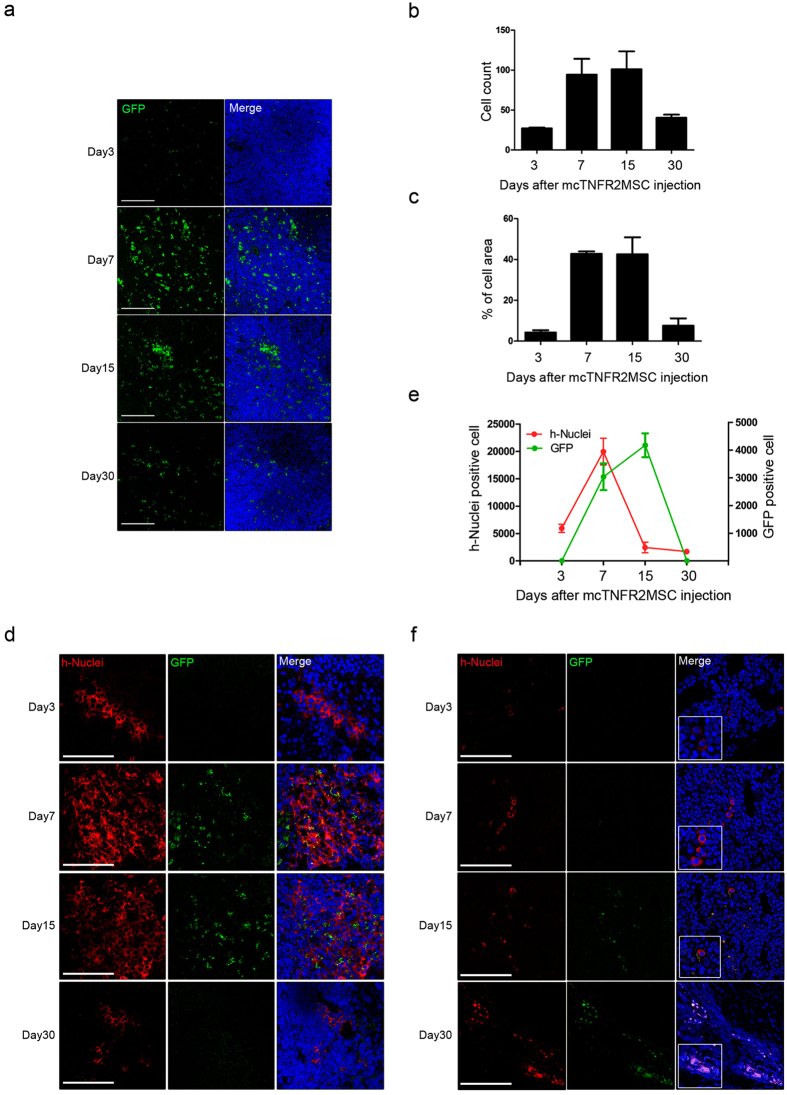
Detection of mcTNFR2MSCs *in vivo*. (**a**) GFP expression of mcTNFR2MSCs in the spleen of CIA mice (n = 5 per time point) is shown by fluorescence. At 3, 7, 15, and 30 days after injection, the spleen was removed from CIA mice and analysed by immunofluorescence staining. Scale bars: 200 μm. (**b**) The number of GFP+ cells was counted in the spleen on each of these days after mcTNFR2MSC injection. (**c**) The percentage of the populated area was calculated by a computer program. (**d**) Time-course analysis of mcTNFR2MSCs in the spleen. At 3, 7, 15, and 30 days after mcTNFR2MSC injection, the spleen was removed from CIA mice and analysed by immunofluorescence staining with both anti-GFP and anti-h-Nuclei antibodies. Scale bars: 200 μm. (**e**) Analysis of numbers of GFP+ and h-Nuclei+ cells. (**f**) Time-course analysis of mcTNFR2MSCs in joints (n = 5 per time point). At 3, 7, 15, and 30 days after mcTNFR2MSC injection, joints were removed from CIA mice and analysed by immunofluorescence staining with anti-GFP and anti-h-Nuclei antibodies. Scale bars: 200 μm.

It has been reported that MSCs mostly migrate into the spleen by inflammatory cytokine-guided homing^30^. Therefore, we screened the spleen to validate the life-span of injected mcTNFR2MSCs *in vivo*. To examine the life-span of mcTNFR2MSCs, CIA mice were sacrificed at 3, 7, 15, and 30 days after the final mcTNFR2MSC infusion. GFP+ cells were frequently observed in the spleen from day 3, and expression of GFP started to increase on day 7 (Fig. 5a). GFP expression increased until day 15; however, it diminished and almost disappeared on day 30. The number of GFP+ cells suddenly increased between day 3 and day 7 (Fig. 5b). GFP+ cells slightly increased after day 7. On day 30, the number of GFP+ cells rapidly decreased to the level seen on day 3. The area of GFP positivity showed a similar pattern (Fig. 5c). Because the injected mcTNFR2MSCs originate from human, they were labelled using a specific antibody against human nuclei (h-Nuclei). h-Nuclei positivity in the spleen was observed only after 3 days (Fig. 5d). Expression was highest on day 7 and this was sustained until day 15. However, expression almost disappeared on day 30. We counted h-Nuclei+ cells and GFP+ cells to confirm the expression tendencies (Fig. 5e). The number of h-Nuclei+ cells started to increase earlier than the number of GFP+ cells (Fig. 5a and d). Expression of h-Nuclei was highest on day 7, which was about 1 week earlier than the peak in GFP positivity. Arthritic joints were also examined to determine if mcTNFR2MSCs migrated to the inflamed region (Fig. 5f). GFP+ cells were detected in joints even on day 3 after the final mcTNFR2MSC injection. Similar to the results for the spleen, GFP signals started to peak later than h-Nuclei signals. However, interestingly, co-localising signals of mcTNFR2MSCs were highly detected in joints on day 30. Based on our results, we conclude that the injected mcTNFR2MSCs populated the spleen after infusion. We confirmed that protein drug synthesis occurs after the migration of MSCs. Moreover, migration of mcTNFR2MSCs was detected in inflamed joints, which can explain the therapeutic effect in CIA mice.

### Anti-inflammatory and anti-osteoclastogenic effects of mcTNFR2MSCs

**Figure 6:**
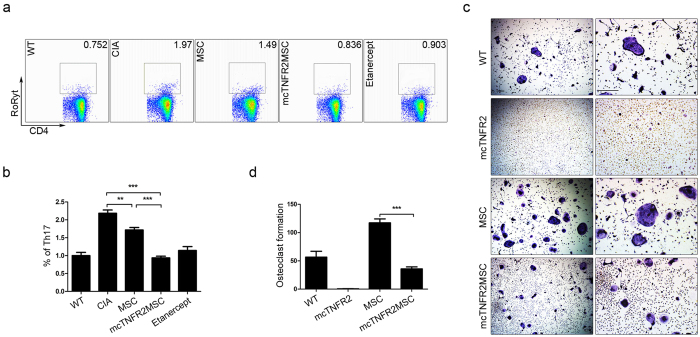
Anti-inflammatory and anti-osteoclastogenic effects of mcTNFR2MSCs transfected with mcTNFR2. (**a**) Splenocytes of mice (n = 5 per group) were analysed by flow cytometry. The RORγt+ population of mcTNFR2MSC-treated mouse splenocytes was similar to that in WT mice. Flow cytometric data were analysed in triplicate. (**b**) Fewer RORγt+ cells were seen in mice administered mcTNFR2MSCs than in mice administered etanercept or MSCs. (**c**) Osteoclast differentiation was induced from WT bone marrow cells (n = 5). Cells were treated with conditioned media of MSCs and HEK293T cells transfected with the minicircle *in vitro*. Scale bars: 200 μm. (**d**) Osteoclasts were counted in three independent experiments. (*P < 0.01; **P < 0.005; ***P < 0.001).

We confirmed that the injected mcTNFR2MSCs tended to migrate to the spleen. We hypothesised that they can influence the population of T lymphocytes in the spleen. Th17 is one of the major cell types that participate in the pathogenesis of RA; therefore, we investigated the population of Th17 cells. The Th17 cell population among splenocytes was analysed by fluorescence-activated cell sorting (Fig. 6a). The population of Th17 cells in the mcTNFR2MSC-injected group tended to decrease to almost half of that in the CIA control group and was similar to that in the WT group. We also confirmed that mcTNFR2MSCs were able to significantly decrease the Th17 cell population compared with WT MSCs (Fig. 6b). Next, we investigated the effect of mcTNFR2MSCs on osteoclastogenesis, which is involved in bone erosion and destruction in RA. Inflammatory cells, particularly the Th17 cell population, promote osteoclastogenesis in arthritis; therefore, we hypothesised that mcTNFR2MSCs would also suppress osteoclastogenesis. As expected, osteoclastogenesis was suppressed by conditioned media of mcTNFR2MSCs (Fig. 6c and d). These results suggest that mcTNFR2MSCs have a superior suppressive effect on the Th17 cell population and osteoclastogenesis compared with MSCs. In conclusion, we confirmed that mcTNFR2MSCs can alter the two major phenotypes involved in the pathogenesis of RA.

## Discussion

MSCs are reported to have immunosuppressive effects against various autoimmune diseases. However, there remain several controversies regarding the therapeutic and immunosuppressive effects of MSCs. Various studies investigated the anti-inflammatory role of MSCs in RA using CIA mice. Djouad *et al*. reported that MSCs do not have any effects in CIA mice^31^. However, various studies report beneficial effects of MSCs in CIA mice^32^,^33^,^34^,^35^,^36^,^37^. The use of various cell strains and differences in the infused cell number or the administration route make it difficult to compare and interpret the inconsistent results of previous studies^38^.

Several groups attempted to modify and improve the curative effects of MSCs because of these controversies. MSCs were transduced with viral vectors, including the genes encoding human proteins^39^ such as CTLA4Ig, BMP2 and IL-10^28^,^39^,^40^,^41^. Even though some viral vectors were confirmed to be safe for clinical use, an improved method that can entirely avoid the integration of viral vectors is required. In this study, we generated mcTNFR2MSCs using minicircle vectors containing the sequence of a human biological drug. The efficacy of minicircle vectors was previously confirmed by several researchers^9^,^10^,^28^,^42^,^43^,^44^. We previously confirmed that minicircle vectors encoding biological drugs (i.e., CTLA4Ig and tocilizumab) can repress the immune reaction in CIA mice^9^,^10^. Mun *et al*. reported that MSCs transfected with minicircles encoding CXCR4 are more likely to migrate to the injury site^44^. However, the effect of minicircle-transfected MSCs on CIA mice has not been reported. To our knowledge, this is the first study to confirm the therapeutic effect of minicircle-transfected MSCs in a RA animal model, namely, CIA.

A variety of cytokines act in the progression and pathogenesis of RA. Among several secreted cytokines, TNFα is thought to be a major player. It is also reported that the coexistence of TNFα can alter the action of MSCs^31^. MSCs are unable to suppress the proliferation of T lymphocytes in allogenic responses in the presence of TNFα. The concentration of an inflammatory cytokine (IL-6) is increased when MSCs are injected in the presence of TNFα. Therefore, MSCs that can block the action of TNFα can be useful for future applications.

Genes can be delivered into MSCs by non-viral methods, including electroporation and chemical transfection. MSCs transfected using Lipofectamine had a high viability; however, the transfection efficacy was lower than that achieved by electroporation. Considering both cell viability and transfection efficiency, we decided to deliver our vector by electroporation. In previous studies of gene delivery into MSCs, gene delivery was poor using chemical transfection, but was about 25–42% using electroporation, which is relatively high^27^. However, with the combination of minicircle vectors and electroporation, we were able to achieve a higher transfection efficacy of about 45–50%. The transfection efficacy was lower than in previous studies using viral vectors; however, it was still slightly higher than that in previous studies using electroporation^24^,^45^. It was recently reported that the microporation of minicircles can increase the transfection efficiency to 63–66%^44^. Mun *et al*. also reported that minicircles can be delivered under milder conditions because of their relatively small size and that cell viability is maintained at up to 96%. The transfection rate is still lower than that achieved using viral delivery methods; however, the integration of viral vectors is one reason why researchers are searching for an alternative tool for transfection.

After transfection, we confirmed that the original characteristics of MSCs were not lost due to the procedure or the soluble drug protein secreted by the minicircles. We also demonstrated that CD marker expression was similar in non-engineered MSCs and mcTNFR2MSCs. However, we cannot conclude that MSCs are exactly the same before and after engineering. We also could not confirm that TNFR2 and commercial etanercept were exactly the same, although the generated TNFR2 bound to an anti-etanercept antibody. This problem may be due to differences between batches of commercial etanercept. We think that the anti-arthritic effects of mcTNFR2MSCs in CIA mice provide indirect evidence of the existence of a synthetic protein similar to commercial etanercept.

Synovial hyperplasia is a major feature of RA. It is associated with pro-inflammatory cytokines including IL-1ß and TNFα^29^,^46^,^47^. Synoviocytes actively proliferate and migrate to adjacent structures. The migration of FLSs is an important pathophysiological phenotype, resulting in joint destruction and consequent dysfunction. Synoviocytes proliferate in a dose-dependent manner when treated with TNFα^48^. The Boyden chamber method was used to demonstrate that TNFα can induce the migration and invasion of FLSs *in vitro*^49^. We tested whether synthetic sTNFR2-Fc inhibited the migration of synoviocytes using an *in vitro* scratch assay. sTNFR2-Fc suppressed the migration of synoviocytes. There are many TNFα-mediated pathological activities other than cell migration. We extended these *in vitro* results to an *in vivo* animal model. The detection of mcTNFR2MSCs was highest between day 7 and day 15 *in vivo*. We also previously confirmed that minicircle expression can be maintained for 10 days. Based on these results, mcTNFR2MSCs were intraperitoneally injected with an interval of 10 days. Injected mcTNFR2MSCs had an anti-arthritic tendency, but there was no statistically significant difference in the arthritic score between mice injected with MSCs and those injected with mcTNFR2MSCs. However, tissue staining showed a remarkable difference between MSCs and mcTNFR2MSCs.

Cytokines and chemokines are associated with MSC migration. Previous studies attempted to show the migration of MSCs; however, divergent results were obtained due to the use of different MSC delivery methods in these studies. It was also reported that MSCs are detected in the spleen after intravenous and intraperitoneal delivery but not in joints using these protocols^16^,^50^,^51^,^52^. In contrast with these previous reports, we detected a small population of mcTNFR2MSCs in joints as well as in the spleen. Migrated human mcTNFR2MSCs expressing GFP were detectable for up to 30 days. The spleen and joints are full of vascular structures and are an important source of chemokines in CIA mice, which might explain why mcTNFR2MSCs tend to migrate into the spleen and joints. Interestingly, h-Nuclei positivity was detected slightly earlier than GFP positivity in the spleen. This can be explained by the initial migration of mcTNFR2MSCs and their subsequent production of TNFR2-Fc. Positive staining of h-Nuclei and GFP in the spleen and joints provides indirect evidence of the detection of mcTNFR2MSCs and sTNFR2-Fc *in vivo*.

Th17 cells and osteoclasts are two major cell types with roles in the pathogenesis of RA^53^,^54^,^55^,^56^. A large number of mcTNFR2MSCs migrated to the spleen; therefore, we investigated the effect of mcTNFR2MSCs on the Th17 cell population among splenocytes. We confirmed the population of RORγt+ cells among splenocytes isolated from mice in each group. Our results correlated with those of a study by Wang *et al*., which confirmed the inhibitory role of MSCs in the regulation of RORγ mRNA and protein expression^57^. Th17 cells stimulate bone resorption by inducing RANKL, which activates osteoclasts^58^. Osteoclasts are induced by treating monocyte-derived macrophages with RANKL *in vitro*. Macrophages are a major cell type that secretes TNFα^59^,^60^. When *in vitro* activated macrophages were treated with the culture supernatant of mcTNFR2MSCs, osteoclast formation was reduced. This inhibitory effect is thought to be due to the blockage of TNFα secretion by monocytes and macrophages, even though there was no additional TNFα treatment. However, the relatively high amount of sTNFR2-Fc secreted by HEK293T cells completely inhibited osteoclast formation. Several studies reported that TNFα synergises the effect of RANKL on osteoclastogenesis^61^,^62^,^63^. Lam *et al*. reported that TNFα can dramatically enhance osteoclast formation, even when RANKL alone cannot^62^. Kobayashi *et al*. also reported that TNFα stimulates gene expression of RANK in osteoclast precursor cells^63^. Therefore, it is thought that the complete inhibition of osteoclasts can be due to relatively increased blockade of TNFα.

MSCs are sometimes dependent on the environment. In an inflammatory environment, MSCs may act as an inflammation agonist and focus on surviving in the harsh conditions. The anti-inflammatory effects of MSCs are reduced or abrogated by their efforts to survive. If we can equip naïve MSCs with anti-inflammatory weapons, they can focus on eliciting anti-inflammatory effects and immune modulation.

In conclusion, etanercept-synthesising MSCs efficiently ameliorated arthritis in an experimental mouse model. Compared with non-engineered MSCs, mcTNFR2MSCs had a superior anti-arthritic activity due to their secretion of etanercept. Although this strategy is at the proof-of-concept stage, it represents a potential alternative method for the delivery of biologics through mcTNFR2MSCs and cell-based therapy.

## Methods

### Cell culture

A bone marrow-derived human MSC (hMSC) line (BM025SS13) was retrieved from The Catholic Institute of Cell Therapy, South Korea. hMSCs were maintained in Dulbecco’s Modified Eagle Medium (DMEM; Gibco Life Technology, Gaithersburg, MD, USA) supplemented with 20% fetal bovine serum (FBS), 100 U/mL penicillin and 100 μg/mL streptomycin (Gibco Life Technology). RA-FLSs were maintained in DMEM supplemented with 10% FBS, 100 U/mL penicillin and 100 μg/mL streptomycin. HEK293T cells were maintained in DMEM supplemented with 7.5% FBS, 100 U/mL penicillin and 100 μg/mL streptomycin. Cells were maintained at 37 °C in 5% CO_2_.

### Production of minicircles

The minicircles (mcMock and mcTNFR2) were produced as described by Kay *et al*.^12^. Briefly, *Escherichia coli* ZYCY10P3S2T cells transformed with the parental plasmids were grown overnight at 37 °C in Terrific Broth containing 50 μg/mL kanamycin. A single colony was grown for 8 h in 2 mL of Luria-Bertani (LB) broth containing kanamycin. The cultures were combined with LB broth containing 0.02% l-(+)-arabinose and incubated at 30 °C for 5 h. Minicircle DNA was isolated using the Nucelobond Xtra Midi kit (Macherey-Nagel, Duren, Germany).

### Minicircle transfection

HEK293T cells were transfected with the minicircle vectors using Lipofectamine 2000 reagent (Invitrogen, Carlsbad, CA, USA) following the manufacturer’s instructions. MSCs at passage 5 were transfected with minicircle vectors using an electroporation system (Neon Transfection System) according to the manufacturer’s instructions. MSCs (5 × 10^5^ cells) were transfected with mcTNFR2 to generate sTNFR2-Fc-expressing cells. Expression of GFP in the cells was assessed by fluorescence microscopy at 48 h post-transfection. The pulse voltage and width conditions were based on the information provided by the Neon Transfection System regarding cell viability and transfection efficacy.

### Detection of sTNFR2-Fc

The conditioned media of transfected HEK293T cells was collected 48 h after transfection. The media was incubated with protein A/G PLUS-agarose beads (Santa Cruz, CA, USA). The captured proteins were detached from the beads and detected by western blotting with an anti-etanercept antibody (Agrisera AB, Sweden). In total, 250ng of Enbrel (AMGEN, CA, USA) in PBS was also loaded onto the gel as a positive control.

### Enzyme-linked immunosorbent assay (ELISA)

To analyse the amount of sTNFR2-Fc protein expressed by transfected cells, the culture media of MSCs transfected with the indicated minicircle vectors was analysed by an enzyme-linked immunosorbent assay (ELISA) at 48 h post-transfection. The levels of sTNFR2-Fc were quantified using a human sTNFR (80 kDa) Platinum ELISA (Affymetrix, San Diego, CA, USA), according to the manufacturer’s instructions. After applying stop solution, absorbance was measured at 450 nm.

### Characterisation of MSC markers

The MSC markers reported by Choi *et al*.^64^ were examined. The immunophenotypes of non-transfected MSCs, mcMockMSCs and mcTNFR2MSCs at passage 5 were determined by flow cytometric analysis. The cells were incubated with an allophycocyanin (APC)-conjugated mouse anti-human CD34 antibody (Affymetrix, 17-0349), a phycoerythrin (PE)-conjugated mouse anti-human CD45 antibody (Affymetrix, 12-9459), a fluorescein isothiocyanate (FITC)-conjugated mouse anti-human CD73 antibody (Affymetrix, 11-0739) and a PE-cyanine 7 (Cy7)-conjugated mouse anti-human CD105 antibody (Affymetrix, 25-1057). The cells were permeabilised using a Foxp3 Transcription Factor Staining Buffer Set (Affymetrix, 00-5523). After cellular staining, cell populations were identified and estimated using an LSR Fortessa Cell Analyser (BD Biosciences, San Jose, CA, USA). The acquired data were analysed using FlowJo V10 single cell analysis software (TreeStar Inc., Ashland, OR, USA).

### Scratch assay of cell migration

The conditioned media of MSCs and HEK293T cells transfected with or without minicircles was collected at 48 h post-transfection. RA-FLSs were seeded onto 6-well plates (Corning-Costar, Tokyo, Japan). The cell monolayer was scratched in a straight line with a P200 pipette tip. Cells were cultured in 2 mL of fresh DMEM supplemented with TNFα (20 ng/mL) or with DMEM alone as a negative control. To examine whether minicircle-derived drugs affect the migration of RA-FLSs, cells were treated with conditioned media of mcTNFR2MSCs for 16 h in the migration assay. In addition, the conditioned media of HEK293T cells transfected with mcTNFR2 and etanercept was used as a positive control and fresh MSC culture media was used as a negative control. Phase-contrast microscopy images were acquired at 0 and 16 h after wounds were created. Using ImageJ software, the invaded area was calculated as follows: percentage of invaded area = (1 − (wounded area at 16 h)/(wounded area at 0 h)) × 100.

### Ethics

All procedures involving animals were in accordance with the Laboratory Animals Welfare Act, the Guide for the Care and Use of Laboratory Animals, and the Guidelines and Policies for Rodent Experimentation provided by the Institutional Animal Care and Use Committee of the College of Medicine of The Catholic University of Korea. This study protocol was approved by the Institutional Review Board of The Catholic University of Korea (CUMC-2015-0084-01).

### Induction of arthritis and evaluation of disease severity

Bovine type II collagen (CII; Chondrex, Redmond, WA, USA) was dissolved in 0.1 M acetic acid to a concentration of 2 mg/mL at 4 °C overnight. CII was emulsified at a ratio of 1:1 with complete Freund’s adjuvant (CFA; Chondrex) containing 2 mg/mL heat-killed *Mycobacterium tuberculosis*. Female DBA1/J mice (n = 5 per group, 6 weeks old; OrientBio, Seongnam, Korea) were intradermally injected at day 0 with 100 mL of the CII/CFA emulsion. The same concentration of CII emulsified with incomplete Freund’s adjuvant (Chondrex) was injected into mice as a booster immunisation 21 days after the first immunisation. Five mice were allocated to the WT group and five mice were each allocated to the CIA, etanercept-treated, MSC-treated and transfected MSC-treated (mcMock and mcTNFR2) groups. The severity of arthritis was monitored and scored as described previously^65^. The severity of inflamed paws was observed for 72 days after the first immunisation.

### *In vivo* delivery of mcTNFR2MSCs

mcTNFR2MSCs (5 × 10^6^ cells) were resuspended in 150 μL of PBS. Cells were delivered by intraperitoneal injection. Cells were injected three times at 20, 30, and 40 days after the first immunisation. Non-engineered WT MSCs and etanercept were used as controls. Mice were administered 25 μg of etanercept (Enbrel; Pfizer, New York, NY, USA) three times per week for 3 weeks after the first immunisation.

### Histological evaluation of arthritis

The hind limbs of each mouse (n = 5 per group) were removed, fixed in 4% paraformaldehyde and decalcified in 10% (w/v) EDTA, and the samples were embedded in paraffin. Sections (4 μm thick) were stained with H&E, safranin O or toluidine blue. The inflammation and joint destruction scores were determined by three individual researchers using the procedure of Huckel *et al*.^66^. The inflammation score of each group was determined according to the severity of infiltration and pannus formation. The destruction score was determined based on cartilage and bone loss.

### Immunofluorescence analysis

Sections of mouse spleen (n = 5 per time point) were fixed in pre-cooled acetone and incubated with 0.3% hydrogen peroxide prepared in 10% methanol for 15 min. After washing with PBS, the sections were blocked using an Avidin/Biotin Blocking Kit (Vector Laboratories, CA, USA). After incubation, blocking was performed following the manufacturer’s instructions of Mouse on Mouse Fluorescein Kit (Vector Laboratories). Sections were incubated with a mouse anti-h-Nuclei monoclonal antibody (Millipore, CA, USA) overnight. The next day, sections were incubated with Alexa Fluor 594-conjugated goat anti-mouse IgG (Invitrogen) for 1 h. After DAPI staining, sections were observed with a confocal microscope (Carl Zeiss LSM700, Prenzlauer, Berlin, Germany).

### Th17 cell population

To detect Th17 cells in the mouse spleen, splenocytes were isolated from each group (n = 5 per group). The cells were incubated with an APC-conjugated rat anti-mouse CD4 antibody (BD Biosciences, 561091) and permeabilised using a Foxp3 Transcription Factor Staining Buffer Set (Affymetrix, 00-5523). The Th17-specific marker RORγt was detected using a PE-conjugated anti-human/mouse RORγt antibody (Affymetrix, 12-6988). After cellular staining, cell populations were identified and estimated using an LSR Fortessa Cell Analyser (BD Biosciences). The acquired data were analysed using FlowJo V10 single cell analysis software (TreeStar Inc.). Cells were first gated for the CD4+ proportion and then analysed for expression of RORγt.

### *In vitro* osteoclast differentiation

Monocytes were isolated from bone marrow of WT mice (n = 5). Samples were treated with ACK buffer to eliminate red blood cells, washed with phosphate-buffered saline and filtered with a strainer. Cells were seeded and maintained in α-MEM supplemented with 10% FBS. The next day, cells were counted, and 3 × 10^5^ cells were stimulated with 10 ng/mL macrophage colony-stimulating factor (M-CSF; Peprotech, Oak Park, CA, USA) for 48 h. Then, a second stimulation was provided by treatment with 30 ng/mL RANKL (Peprotech) and M-CSF. Cells were treated with conditioned media of transfected MSCs or HEK293T cells, adding the conditioned media of non-transfected MSCs as required. After 72 h, differentiated osteoclasts were stained for TRAP following the manufacturer’s guide. Stained cells were counted.

### Statistical analysis

All experiments were repeated three or more times. The results are shown as mean and standard error of the mean. Error bars represent the standard error of the mean. Statistical analysis was performed and graphs were drawn using GraphPad Prism 5 (GraphPad). The T-test was applied to analyse non-parametric quantitative datasets, and the one-tailed p-value was calculated. *P < 0.01; **P < 0.005; and ***P < 0.001 indicated statistical significance.

## Additional Information

**How to cite this article**: Park, N. *et al*. Etanercept-Synthesising Mesenchymal Stem Cells Efficiently Ameliorate Collagen-Induced Arthritis. *Sci. Rep.* **7**, 39593; doi: 10.1038/srep39593 (2017).

**Publisher's note:** Springer Nature remains neutral with regard to jurisdictional claims in published maps and institutional affiliations.

## Supplementary Material

Supplementary Figure S1
