# Supplementary material for: Etanercept-Synthesising Mesenchymal Stem Cells Efficiently Ameliorate Collagen-Induced Arthritis
Source: Sci Rep. 2017 Jan 13;7:39593. doi: 10.1038/srep39593 (PMC5234034; doi:10.1038/srep39593)
Supplement: Supplementary Figure S1 [file srep39593-s1.pdf]

**Supplementary Information**

**Etanercept-Synthesising Mesenchymal Stem Cells Efficiently Ameliorate Collagen-Induced Arthritis.**

**Authors:** Narae Park[1], Yeri Alice Rim[1], Hyerin Jung[1], Juryun Kim[1], Hyoju Yi[1,2], Youngkyun Kim[1,2], Yeonsue Jang[1], Seung Min Jung[1], Jennifer Lee[1], Seung-Ki Kwok[1], Sung-Hwan Park[1], and Ji Hyeon Ju[1] *

**Affiliations:**

1. Division of Rheumatology, Department of Internal Medicine, Seoul St. Mary's Hospital, College of Medicine, The Catholic University of Korea, Seoul, 137-701, Republic of Korea.

2. Department of Medicine, Institute for Stem Cell Biology and Regenerative Medicine, and Stanford Cardiovascular Institute, Stanford University School of Medicine, Stanford, CA, USA.

***Corresponding author.**

Ji Hyeon Ju, M.D., Ph.D.

Division of Rheumatology, Department of Internal Medicine, Seoul St. Mary's Hospital, College of Medicine, The Catholic University of Korea, Banpodaero 222, Seochogu, Seoul, 137-701, Republic of Korea, Tel: 82-2-2258-6893, Fax: 82-2-3476-2274, E-mail: juji@catholic.ac.kr.

## Supplementary figures

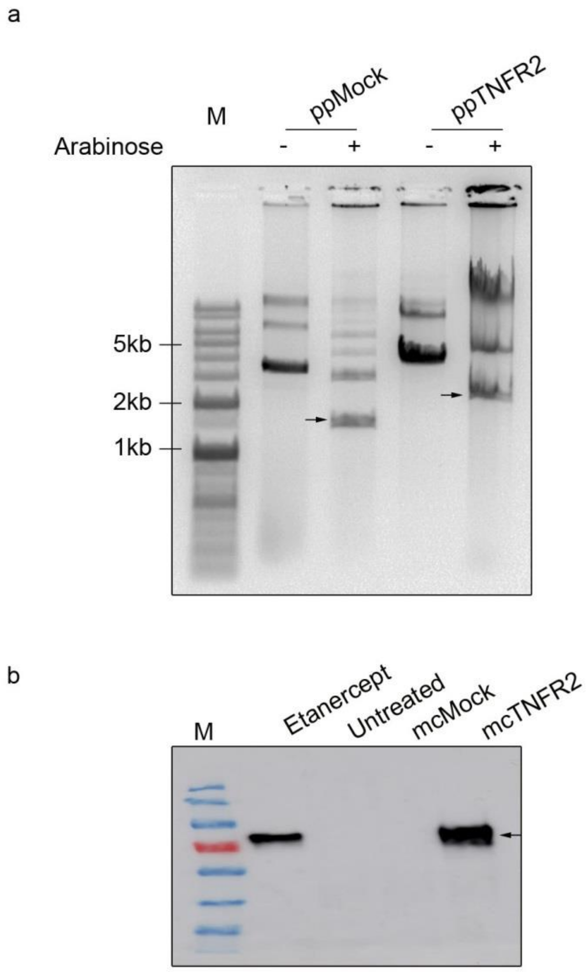


**Supplementary Figure S1.** Generation and drug expression of the mcTNFR2. (a) The full length gel electrophoresis of parental plasmids (mock and sTNFR2-Fc) after arabinose incubation. Minicircles with an appropriate size were detected after arabinose induction (arrows). (b) The full length western blot of sTNFR2-Fc expression. sTNFR2-Fc was detected in the conditioned media of mcTNFR2-transfected HEK293T cells (arrows). Etanercept was used as a control.
